# Fiber Ring Laser Directional Torsion Sensor with Ultra-Wide Linear Response

**DOI:** 10.3390/s19163613

**Published:** 2019-08-20

**Authors:** Xianjin Liu, Fengjuan Wang, Jiuru Yang, Xudong Zhang, Xiliang Du

**Affiliations:** 1 College of Electronics Engineering, Heilongjiang University, Harbin 150080, China; 2 School of Electronic Information and Electrical Engineering, Chongqing University of Arts and Sciences, Chongqing 402160, China

**Keywords:** fiber laser sensor, Sagnac interferometer, torsion, linear response range, phase modulation

## Abstract

In this paper, a comprehensive passive torsion measurement is performed firstly in a 40-cm-long polarization maintaining fiber-based Sagnac interferometer (PMF-SI), and the non-linear torsion response is found and investigated. Then, a fiber laser torsion sensor (FLTS) with a dual-ring-cavity structure is proposed and experimentally demonstrated, in which the PMF-SI is utilized as the optical filter as well as the sensing unit. In particular, the highly sensitive linear range is adjusted through fine phase modulation, and owing to the flat-top feature of fringes, an ~83.6% sensitivity difference is effectively compressed by the generated lasing. The experimental results show that, without any pre-twisting, the ultra-wide linear response from –175 to 175 rad/m is gained, and the torsion sensitivities are 2.46 and 1.55 nm/rad with high linearity (>0.99) in the clockwise and anti-clockwise directions, respectively. Additionally, a high extinction ratio (>42 dB) and small line-width (~0.14 nm) are obtained in the proposed FLTS, and the corresponding detection limit reaches 0.015 rad/m.

## 1. Introduction

To date, fiber torsion sensors have played indispensable roles in aerospace engineering, artificial intelligence, and industrial automation due to the merits of their compact size, high sensitivity, low cost, and immunity to electromagnetic interference. Lots of schemes have been demonstrated based on fiber gratings [1,2,3,4,5,6], modal interferometers [7,8,9,10], Sagnac interferometers (SI) [11,12,13,14,15,16,17,18], microfibers [19,20], and multi-/hollow-core fibers [21,22,23]. Among them, polarization-maintaining fiber (PMF) based Sagnac-loop structures are viewed as one of the most practical torsion sensors owing to their stability and cost-efficiency. To enhance sensitivity, the etched and heated PMFs were respectively adopted in [14,16], and the maximum sensitivity reached 18.60 nm/(rad/m). Furthermore, Huang reported a low-birefringence single-mode fiber (SMF) based SI and the highest sensitivity of up to 3.25nm/° was exhibited in the range from −10 to 80 degrees [17]. Nevertheless, it is worth noting that most SI-based torsion sensors show non-linear responses to the varied torsion, typically a sine- or sinc-function [12,17,18,24]. That means that pre-twisting is usually necessary for direction discrimination. In addition, for the passive schemes, a common issue is that the detection limit of sensors is constrained by the low visibility and large line-width (LW) of fringes.

Alternatively, fiber laser sensors (FLS) with a high extinction ratio (ER) and small LW have received much attention as an effective method for improving detection limits. Additionally, FLS-based schemes have been continuously applied in the sensing of temperature, refractive index (RI), strain, and curvature [25,26,27,28,29,30,31,32]. Recently, Shi *et al*. reported a fiber ring laser for torsion sensing using cascaded helical long-period gratings (HLPG), and an ultra-wide linear response of torsion from −100 to 100 rad/m was presented but with a sensitivity of 0.084 nm/(rad/m) [33]. Díaz et al. demonstrated a distributed Bragg reflector laser torsion sensor based on frequency modulation [34].

In this paper, a novel dual-ring-cavity-based FLS for directional torsion measurement is proposed and experimentally demonstrated, in which a PMF-based Sagnac loop structure is utilized as an optical filter as well as the sensing unit. Moreover, owing to the flat-top feature of fringes, an ~83.6% sensitivity difference is effectively reduced by the generated lasing. Therefore, under fine phase modulation, an ultra-wide linear response from −175 to 175 rad/m is gained with a linearity of 0.991. Additionally, the torsion sensitivities reach 2.46 and 1.55 nm/rad in the clockwise (CW) and anti-clockwise (ACW) directions, respectively. Additionally, the proposed FLTS has an ER of >42 dB and an LW of ~0.14 nm, and the corresponding detection limit reaches 0.015 rad/m. In addition, the proposed FLTS is ease of fabrication, cost-efficiency, and has the potential for application in structural health monitoring and industrial automation.

## 2. Principles

**Figure 1:**
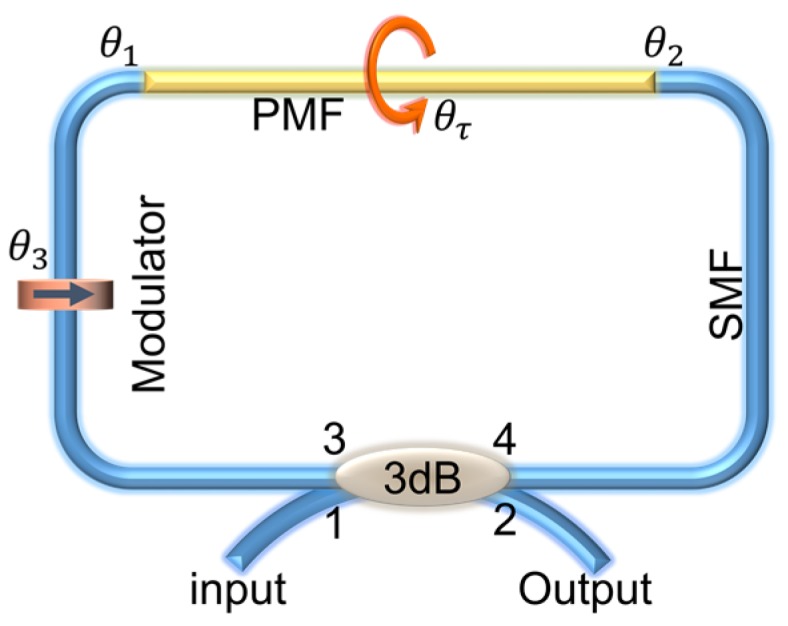
Schematic diagram of the Sagnac interferometer.

In a Sagnac loop, as shown in Figure 1, the input light is equally split into two propagation beams and re-combined through a 3 dB coupler, which corresponds to the orthogonal polarization modes $HE_{11}^{X}$ and $HE_{11}^{Y}$ propagating along the fast and slow axes of PMF. Therefore, a stable interference will be formed because of the refractive index (RI) difference between $HE_{11}^{X}$ and $HE_{11}^{Y}$. Furthermore, we define $\theta_{1}$ and $\theta_{2},$ respectively, as denoting the angles between the light at the spliced point and the fast axis of PMF, and $\theta_{3}$ symbolizes the phase shift caused by a modulator.

According to [35], when ignoring the insertion loss, the transmission spectrum of PMF-SI is derived as

(1) $$T={\left[\sin\left(\theta_{1}+\theta_{2}\right)\cos\left(\frac{\varphi }{2}+\theta_{3}\right)\right]}^{2}$$

where φ is the phase difference caused by $HE_{11}^{X}$ and $HE_{11}^{Y}$, and it can be written as

(2) $$\varphi =\frac{2\pi \left(n_{s}-n_{f}\right)L}{\lambda }=\frac{2\pi BL}{\lambda }$$

where *λ* is the wavelength of incident light, *L* is the length of the PMF, $n_{s}$ and $n_{f}$ are the RI of the PMF fast and slow axes, and $B=n_{s}-n_{f}$. For the given angles of $\theta_{1}$, $\theta_{2},$ and $\theta_{3}$, Equation (1) can be simplified as

(3) $$T\approx \frac{1-\cos\left(\varphi \right)}{2}$$

When φ=2mπ (*m* is an integer), the resonance wavelength $\lambda_{dip}$ will be

(4) $$\lambda_{dip}=\frac{BL}{m}$$

and the free spectrum range (FSR) is approximately written as

(5) $$FSR\approx \frac{\lambda^{2}}{BL}$$

Further, when the PMF is twisted, its effective RI change caused by shear strain will be expressed as

(6) $$\Delta n_{s}=g_{s}\tau n_{s}$$

$$\Delta n_{f}=g_{f}\tau n_{f}$$

where $g_{s}$ and $g_{f}$ are the photo-elastic coefficients of the fast and slow axes, respectively. *τ* = *θ_τ_* /*l* is the twist rate, where $\theta_{\tau }$ is the twist angle and *l* is the length of twisted fiber. Assuming $\Delta B=\Delta n_{s}-\Delta n_{f}$, the phase change Δφ will be written as

(7) $$\Delta \varphi =\frac{2\pi }{\lambda }\left(\Delta LB+\Delta Bl\right)$$

Neglecting the effect of longitudinal strain (ΔL→0), Equation (7) is changed to $\Delta \varphi =\frac{2\pi }{\lambda }\Delta Bl$. Combined with Equation (2), the wavelength shift can be derived as

(8) $$\Delta \lambda =\frac{\left(g_{s}n_{s}-g_{f}n_{f}\right)\theta_{\tau }}{BL}\lambda_{dip}.$$

Equation (8) shows that Δλ is merely proportional to $\theta_{\tau }$ for a given *L,* and the torsion direction can be recognized by the red- or blue-shift of $\lambda_{dip}$. However, due to the varied $g_{s}$ and $g_{f}$ under the added shear strain, the value of Δλ is usually a non-linear response to the applied torsion. This means a PMF-SI based torsion sensor may be only effective in a small range and needs a pre-twisting angle to assist direction discrimination. Our work then focuses on enlarging the linear response region of torsion under the non-pre-twisting state.

## 3. Experiments and Results

**Figure 2:**
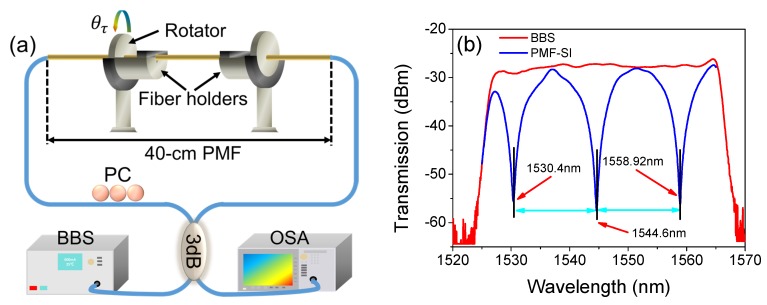
(**a**) The experimental setup for passive torsion-sensing and (**b**) the transmission spectra of BBS and PMF-SI. PMF: polarization-maintaining fiber, PC: polarization controller BBS: broadband source, OSA: optical spectrum analyzer, SI: Sagnac interferometer.

As shown in Figure 2a, the Sagnac loop structure was mainly formed by a 40 cm long PMF (CETC, PM1550-1-06002-3) and a 3 dB SMF-coupler. The input and output ports of the coupler respectively connected with a broadband light source (BBS, homemade, operated in the range of 1525–1565 nm) and an optical spectrum analyzer (OSA, Agilent 86142B, with a resolution of 0.01 nm). The PMF was straightly fixed on the fiber holder and rotator with a small axial stress in order to reduce the fiber bending effect. A polarization controller (PC) was used as a modulator to introduce the matched phase change. Additionally, the distance between two holders was fixed at ~30 mm in order to get a large torsion range. Figure 2b shows the transmission spectra of the BBS (red-line) and PMF-SI (blue-line). The BBS had a flat spectrum with fluctuations of less than 1.9 dB across the whole 40 nm bandwidth, and the three formed fringes were located at 1530.4, 1544.6, and 1558.92 nm. Moreover, the average visibility reached about 25.6 dB with approximately the same FSR (~14.3 nm).

**Figure 3:**
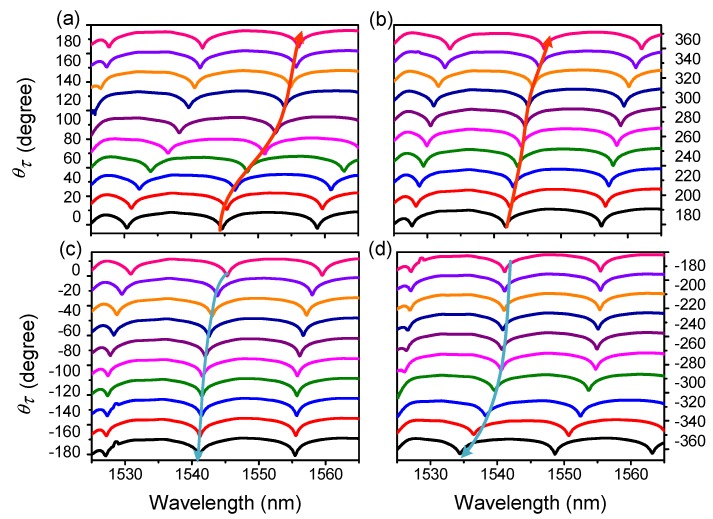
Spectra evolution of PMF-SI in the clockwise (CW) direction from (**a**) 0° to 180° and (**b**) 180° to 360° and the anticlockwise (ACW) direction from (**c**) 0° to −180° and (**d**) −180° to −360°.

**Figure 4:**
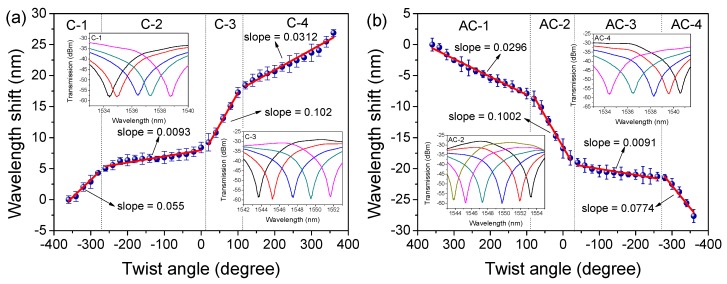
Relationships between the wavelength shift and twist angle in (**a**) the clockwise direction and (**b**) the anticlockwise direction.

Comprehensive torsion measurements were then performed with the varied $\theta_{\tau }$ in the range from −360° to 360° with an interval of 20°. According to Figure 3a,b, it is clear that the wavelength red-shifted in the CW direction from 0° to 360°, but blue-shifted in the ACW direction from 0° to −360°, as shown in Figure 3c,d. Nevertheless, we also noticed that this wavelength shift was non-linear. Figure 4 shows the torsion response in the CW (from −360° to 360°) and ACW (from 360° to −360°) directions, and the quasi-periodic feature of wavelength shift is exhibited with the added $\theta_{\tau }$.

For clarity, the sensitive and insensitive regions in Figure 4a,b were divided into four parts, denoted by C-*i* and AC-*i* (*i* = 1~4), respectively. In more detail, as shown in Figure 4a, the highly sensitive regions were located at C-1(approximately –360° to –260°) and C-3 (0°~120°) with sensitivities of 102 and 55 pm/°. However, the sensitivities were merely ~9.3 and 31.2 pm/° in their neighboring regions C-2 (approximately –260° to 0°) and C4 (120°~360°). These results mean that the PMF-SI is only sensitive to the varied torsion in a small range (100°~120°). Moreover, the maximum sensitivity difference (SD) at around 0° reached 92.7 pm/°, and the minimum value between C-1/C-3 and C-2/C-4 was ~22 pm/°. Similar results were also observed in the ACW direction and are presented in Figure 4b. By calculation, the maximum SD (between AC-2 and AC-3) was found to be 91.1 pm/°, and the minimum SD (between AC-1/AC-3 and AC-2/AC-4) was 21 pm/°. Such high SD values indicate that a pre-twisting angle may be necessary to complete an effective directional torsion measurement.

**Figure 5:**
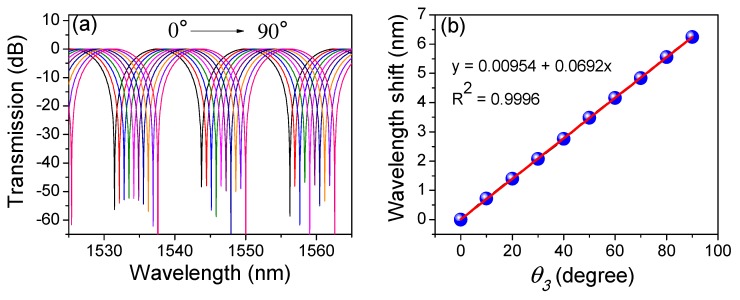
(**a**) Numerical simulation of the spectral evolution with the varied $\theta_{3}$ and (**b**) the relationship between wavelength shift and $\theta_{3}$

**Figure 6:**
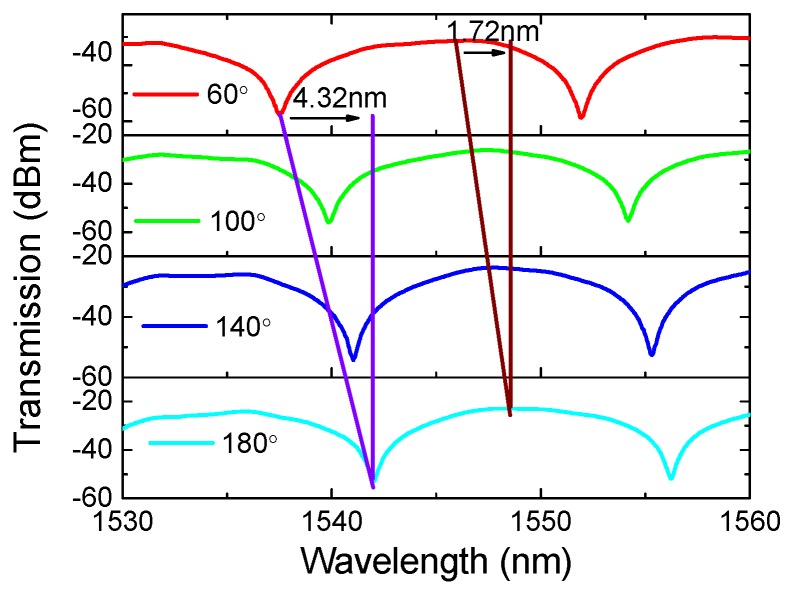
Interference fringe peak and dip wavelength shift with the changed $\theta_{\tau }$.

Equation (1) shows that the transmission spectrum of PMF-SI can be adjusted by $\theta_{3}$. A numerical simulation was conducted to reveal the relation between Δλ and $\theta_{3}$. As shown in Figure 5a, the interference fringes red-shifted with the varied $\theta_{3}$ in the range from 0 to $\frac{\pi }{2}$. Additionally, Figure 5b shows that Δλ linearly increased with the added $\theta_{3}$ and the coefficient was 0.0692 nm/°. In [36], it was shown that $\theta_{3}$ can be adjusted and implemented by an external modulator, such as a PC. Additionally, the wavelength-shift difference between the dips and peaks of fringes was investigated. As shown in Figure 6, the dip-shift was about 4.32 nm but the peak merely shifted by 1.72 nm when $\theta_{\tau }$ = 60°~180°. This flat-top feature means that the high SD presented in Figure 4 will be greatly reduced and homogenized when the peaks of fringes are utilized. Additionally, the linear response range will definitely be enlarged although a ~60% reduction in torsion sensitivity will occur.

**Figure 7:**
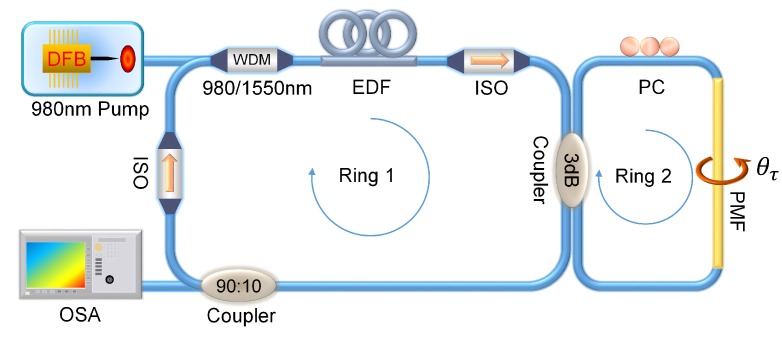
The schematic diagram of the fiber laser torsion sensor (FLTS). WDM: wavelength division multiplexer, EDF: erbium-doped fiber, ISO: isolator.

The above analysis indicates that a torsion sensor with an ultra-wide linear response can be achieved by combining a fiber ring laser (FRL) and a well-phase-modulated Sagnac-loop. The experimental setup of fiber laser torsion sensor (FLTS) with a dual-ring-cavity structure is shown in Figure 7, where the Sagnac loop (denoted by Ring-2) as an optical filter as well as the sensing unit is connected with the FRL (denoted by Ring-1) by a 3 dB coupler. In Ring-1, a laser diode (LD, Oclaro LC96HH74P-20R) with a central wavelength of 976 nm is used as the pump source. Through a 980/1550 nm wavelength division multiplexer, a 10 m long erbium-doped fiber (EDF, Nufern EDFC-980-HP) was stimulated by amplified spontaneous emission. Then, the generated broadband light with the band of 1525–1565 nm was equally split into Ring-2.

**Figure 8:**
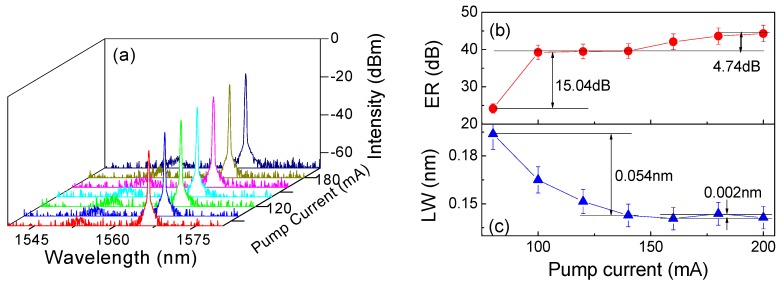
(**a**) The output spectra, (**b**) extinction ratio (ER) and (**c**) line width (LW) of the fiber laser ring (FRL) under a varied pump current (from 80 to 200 mA).

By fine adjustment of the PC, the output from the Sagnac-loop was stably operated in Ring-1 and the formed lasing was finally monitored and recorded by OSA. In particular, two isolators were used to prevent the effects of reflection and spatial hole-burning. Figure 8a shows the output spectra of FRL under varied pump current (denoted by *I_P_*) in the range of 80~200 mA. Clearly, with the rise of *I_P_*, the output intensity of lasing continuously increased and the maximum intensity located at 1566.13 nm reached −18.23 dBm when *I_P_* *=* 200 mA. Furthermore, the relationship between the ER/LW of lasing and *I_P_* was investigated. Figure 8b shows an obvious rise in ER (~15.04 dB) when *I_P_* increased from 80 to 100 mA. However, the rise greatly decreased in the range of 100~200 mA to merely ~4.74 dB. Similarly, Figure 8c shows that the LWs of lasing quickly decreased from 0.19 to 0.14 nm when *I_P_* increased from 80 to 140 mA. However, the LW values were maintained at ~0.14 nm when *I_P_* > 140 mA. Considering the stability of FRL, *I_P_* *=* 160 mA was selected and the corresponding LW and ER of lasing were 0.14 nm and 42.18 dB, respectively.

**Figure 9:**
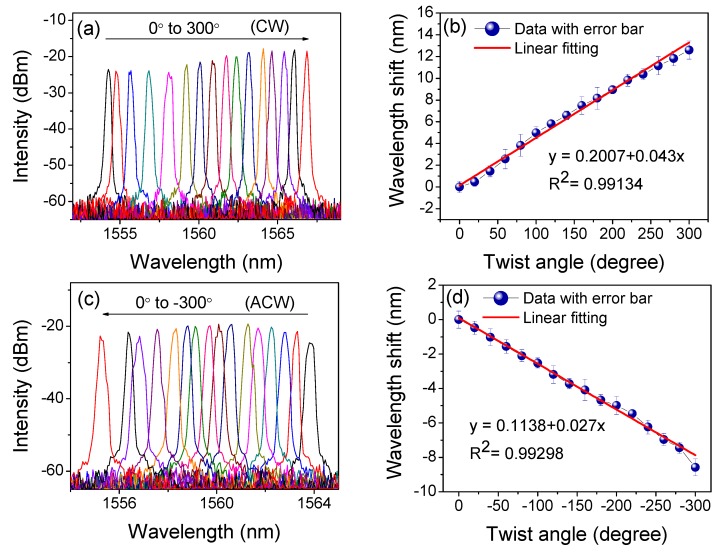
(**a**) Twist-induced lasing-wavelength shift and (**b**) response in the CW direction; (**c**) Twist-induced lasing-wavelength shift and (**d**) response in the ACW direction.

**Figure 10:**
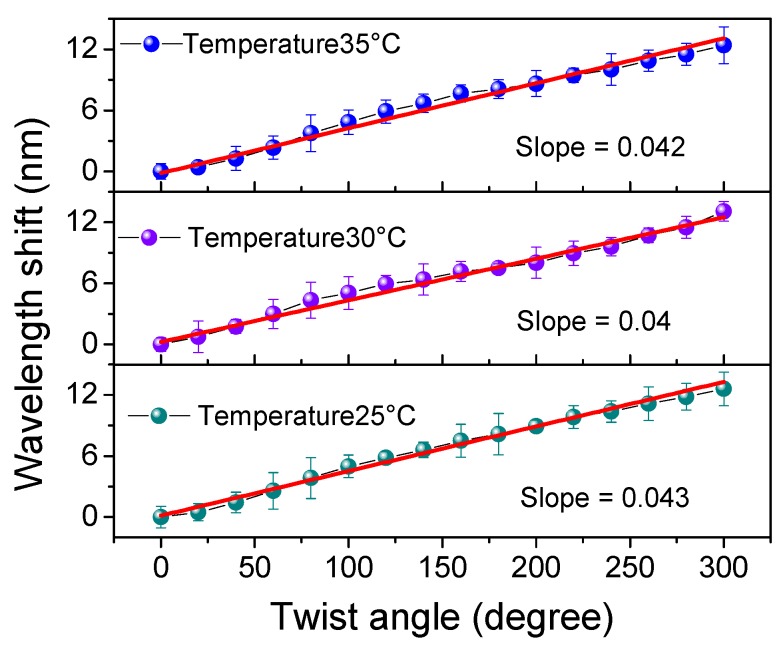
The torsion responses with varied ambient temperature.

We then set *I_P_* = 160 mA and twisted the PMF with the varied $\theta_{\tau }$. As shown in Figure 9a, the emitted lasing red-shifted by about 12.4 nm in the CW direction with a fluctuation of 6.57 dB. Additionally, the relationship between Δλ and $\theta_{\tau }$ is shown in Figure 9b. Compared to the results in Figure 4a, the SD was obviously compressed in the range from 0° to 300° (which corresponds to the range of 0~175 rad/m). Thus, the calculated torsion sensitivity reached 2.46 nm/rad (43 pm/°) with a linearity of 0.991. Similar results in the ACW direction are demonstrated in Figure 9c,d but with a blue-shift in wavelength. By calculation, the torsion sensitivity in ACW direction was determined to be 1.55 nm/rad (27 pm/°) with a linearity of 0.993 in the range from 0° to −300° (which corresponds to the range of approximately 0 to −175 rad/m). Compared to the results shown in Figure 4, the sensitivity in FLTS was reduced by about 57% in the CW direction and 63.5% in the ACW direction, respectively. However, the SD was also reduced by ~82.6% (=$1-\frac{43-27}{\left(92.7+91.1\right)/2}$) across the whole range of −300° to 300°. We then measured the repeatability of our FLTS at different temperatures, and the corresponding relationships between the wavelength and twist angle are given in Figure 10. According to the measured results, similar torsion sensitivities (~0.04 nm/°) were presented in the range from 0° to 300°. So, the maximum SD at different temperatures was restrained within 0.003 nm/°. This tiny value of SD indicates that our sensor has a stable wavelength response in torsion with a varied ambient temperature.

A comparison between our work and other related torsion sensors is given in Table 1. It is obvious that the largest linear range, ±300° (±175 rad/m), was obtained with our proposed FLTS, which is an improvement of 2.5~10 times compared with the listed passive schemes. It is worth noting that, as mentioned above, this wide linear range was gained with a more than 50% reduction in sensitivity. Fortunately, according to Equation (8), the torsion sensitivity of FLTS can be enhanced by simply reducing the length of the PMF.

**Figure 11:**
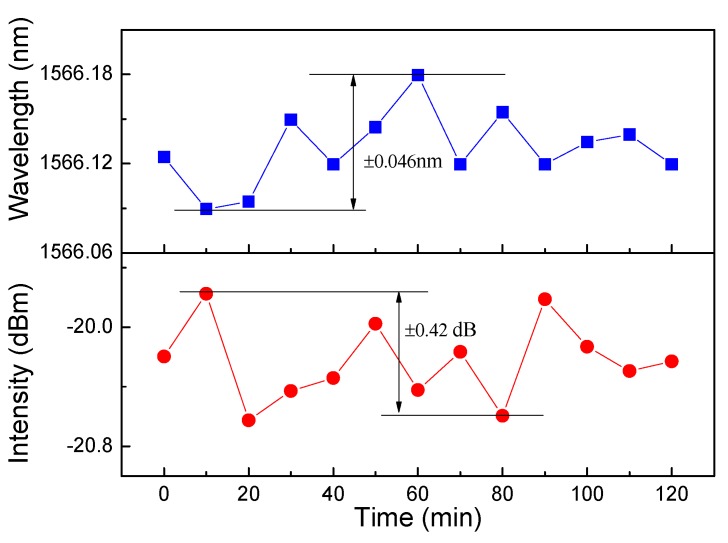
The stability of laser wavelengths within 2 hours.

Furthermore, the detection limit of FLTS is defined by

(9) $$DL=\frac{R}{S}$$

where *S* is the torsion sensitivity and *R* is the detection resolution. From [30], *R* can be expressed as

(10) $$R=3\sqrt{\sigma_{ampl-noise}^{2}+\sigma_{temp-noise}^{2}+\sigma_{spect-res}^{2}}$$

where $\sigma_{ampl-niose}$, $\sigma_{temp-noise}$ and $\sigma_{spect-res},$ respectively, denote the errors caused by amplitude noise, thermal variation, and spectral resolution. In general, $\sigma_{ampl-niose}$ is small enough to be ignored at room temperature and $\sigma_{spect-res}=R_{W}/2\sqrt{3}$, where $R_{w}$ is the wavelength resolution of OSA. $\sigma_{ampl-niose}=\frac{LW}{4.5{\left(\mathrm{ER}\right)}^{0.25}}$ is the main noise and is dependent on the values of LW and ER. In our sensor, the calculated $\sigma_{ampl-niose}$ was equal to 1.25 × 10^-2^ nm and $\sigma_{spect-res}$ was 2.89 × 10^-3^ when the parameters LW = 0.14 nm, ER = 42.18 dB, and $R_{w}$ = 0.01 nm were adopted. The corresponding resolution was 0.0376 nm and the DL of FLTS reached 0.015 rad/m. In addition, a stability test of the output lasing was performed with respect to the wavelength and intensity when *I_p_* =160 mA and the room temperature was kept at 25 ± 0.2 ℃. Figure 11 shows that within 2 hours, the wavelength and intensity fluctuations of FLTS were constrained within ±0.046 nm and ±0.42 dB, respectively. 

## 4. Conclusions

In this paper, the flat-top feature of the fringes of PMF-SI was investigated and a novel dual-ring-cavity based FLTS was proposed and completed in order to reduce the non-linear response of torsion. Through fine phase modulation, the torsion response curve shifted and homogenized in the FLTS. The experimental results showed that the sensitivity difference was effectively compressed and an ultra-wide linear response from −175 to 175 rad/m was gained. Moreover, the acceptable torsion sensitivities were 2.46 and 1.55 nm/rad with a linearity of 0.99 in the CW and ACW directions, respectively. The corresponding detection limit reached 0.015 rad/m owing to the high ER and small LW. Additionally, the wavelength and intensity fluctuations of FLTS were limited to ±0.09 nm and ±0.42 dB within 2 hours. The proposed FLTS is stable, practical, and very promising for the applications of structural health monitoring and industrial monitoring.

## Figures and Tables

**Table 1:** Comparisons of fiber torsion sensors. HPLG: helical long-period gratings. SMF: single-mode fiber.

Structures	Linear Response Range	Sensitivity	Direction	Refs.
photonic crystal fiber-based SI	approx. −90 to 90 deg	0.059 nm/deg	Yes	[13]
elliptical-core PMF-based SI	approx.−120 to 120 deg	0.68 nm/deg	Yes	[14]
low birefringence SMF-based SI	180~270 deg	3.26 nm/deg	No	[17]
tapered seven-core fiber	540~640 deg	0.88 nm/deg	Yes	[22]
reflective Lyot filter	10~50 deg	20.336 dB/rad	No	[24]
quasi-fan Solc filter	40~90 deg	1.27 dB/(rad/m)	No	[37]
HLPG-based FLTS	−100 to 100 rad/m	0.084 nm(rad/m)	Yes	[33]
SI-based FLTS	±300 deg (±175 rad/m)	2.46 nm/rad	Yes	This work
